# Keeping track of mosquitoes: a review of tools to track, record and analyse mosquito flight

**DOI:** 10.1186/s13071-018-2735-6

**Published:** 2018-03-02

**Authors:** Jeroen Spitzen, Willem Takken

**Affiliations:** Laboratory of Entomology, Wageningen University and Research, PO Box 16, 6700 AA Wageningen, The Netherlands

**Keywords:** Flight behaviour, Automated tracking, Mosquito, 3D analysis, Infectious diseases, Malaria, Behavioural ecology, Vector control

## Abstract

The health impact of mosquito-borne diseases causes a huge burden on human societies. Recent vector control campaigns have resulted in promising declines in incidence and prevalence of these diseases, notably malaria, but resistance to insecticides and drugs are on the rise, threatening to overturn these gains. Moreover, several vector-borne diseases have re-emerged, requiring prompt and effective response measures. To improve and properly implement vector control interventions, the behaviour of the vectors must be well understood with detailed examination of mosquito flight being an essential component. Current knowledge on mosquito behaviour across its life history is briefly presented, followed by an overview of recent developments in automated tracking techniques for detailed interpretation of mosquito behaviour. These techniques allow highly accurate recording and observation of mating, feeding and oviposition behaviour. Software programmes built with specific algorithms enable quantification of these behaviours. For example, the crucial role of heat on host landing and the multimodal integration of carbon dioxide (CO_2_) with other host cues, has been unravelled based on three-dimensional tracking of mosquito flight behaviour. Furthermore, the behavioural processes underlying house entry and subsequent host searching and finding can be better understood by analysis of detailed flight recordings. Further potential of these technologies to solve knowledge gaps is discussed. The use of tracking techniques can support or replace existing monitoring tools and provide insights on mosquito behaviour that can lead to innovative and more effective vector-control measures.

## Background

Mosquito-borne diseases continue to impose a heavy burden on human societies and impede welfare and economic development [1]. Despite promising declines of malaria incidence within the last 15 years [2], other mosquito-borne diseases such as Zika, dengue, chikungunya and West-Nile virus are on the rise and have spread over various continents [3, 4]. In addition to climate change, international trade and human transport are considered to be the main drivers of introductions of new vectors, with or without their pathogens, into different geographical regions [5–9].

The recently reported global decline in malaria was achieved mainly by wide-spread use of long-lasting insecticide-treated nets (LLINs), indoor residual spraying (IRS) and proper drug treatment, but insecticide resistance and resistance to antimalarial drugs threaten to prevent further reductions or may even lead to disease resurgence [10, 11]. Innovative alternative methods are being developed, such as the use of entomopathogenic fungi [12], biolarvicides (*Bacillus* spp.) [13, 14], push-pull systems and mass trapping techniques using odour-baited traps [15, 16] in combination with house improvements [17]. It is expected that the combined use of these tools, along with LLINs, IRS and proper drug treatment, may provide a more sustainable strategy for vector-borne disease control [18]. Recently, the World Health Organization launched a strategic approach named Global Vector Control Response, aiming for locally adapted sustainable control measures to target multiple vectors [19, 20].

Successful implementation of new vector control tools along with existing tools following the integrated vector management approach requires detailed understanding of mosquito behaviour. For example, the contact rate of mosquitoes with insecticide-treated bed nets is an important parameter in understanding the efficacy of this technology [21, 22]; higher efficacy of mosquito traps requires knowledge on trap entry behaviour [23–25], and push-pull strategies can be made more effective if mosquito foraging behaviour in the peri-domestic area is well understood [26].

Innovative tracking techniques have opened a new array of possibilities for examining insect behaviour in both the laboratory and (semi-) field [27–29]. The focus of our review is on the tracking of mosquitoes in space in order to elucidate fundamental aspects of their behaviour in different phases of their life history. An overview of tools used for behavioural tracking of mosquitoes and their technical complexities is provided. We discuss how in-depth knowledge on mosquito behaviour can be exploited for the development and evaluation of vector control strategies.

## Behaviour across the mosquito life history

The mosquito life history traits can be divided into plant feeding, mating, host feeding, oviposition, larval development and pupation. There is surprisingly little fundamental knowledge about the behavioural aspects of these phases, despite a wealth of knowledge on the factors affecting these behaviours [30–36]. The behavioural aspects of these phases are briefly described below.

We pay attention to anthropophilic mosquito species which forage in and around human dwellings in search of a blood meal [37]. For *Anopheles* species, many of these behaviours occur in the evening or at night, when darkness makes direct observations more challenging. Video-recording makes it possible to visualize mosquito behaviour on a small scale, even during the scotophase, without having the experimental set-up affected by (extra) cues associated with the researcher. Besides host-seeking, describing other behaviours during the mosquito life-cycle, e.g. mating, (post-) feeding and oviposition, can also greatly benefit the understanding of mosquito-borne disease ecology and further assist in the development and evaluation of surveillance and intervention tools.

### Plant feeding

Among the first activities of a mosquito after emergence from the pupa is the search for a sugar source. Carbohydrates provide energy for mosquitoes’ daily requirements and males rely fully on sugar-feeding [38]. The sugar source is mostly nectar, but can also consist of fruits, honeydew or extra-floral nectar. Mosquitoes can show strong preferences for certain plant sources (reviewed by [39]). Deprivation of sugar sources affects the flight capacity and can consequently affect mosquito dispersal, mating success and/or host-finding [40–42].

Recent studies indicate that mosquitoes can learn to associate visual cues with the quality of sugar sources [30]. Such studies contribute to further development of effective use of (toxic) sugar baits to manipulate or control mosquito vectors [43, 44] or to monitor the presence of pathogens in local populations [45, 46]. Behavioural studies on sugar-feeding can contribute to the knowledge of preferred plant sources, time-budget spent based on efficiency and duration of feeding and possible competition for these sugar sources with other organisms visiting the plant [31, 38]. In addition, questions such as whether mosquitoes are important for pollination can be answered based on behavioural observations; this is an area of research showing growing evidence that mosquitoes are not just nectar thieves [38, 47, 48].

### Mating

Mosquitoes, along with other Diptera, have the special ability to mate in flight [49]. Depending on the species, mating occurs in swarms formed by males (most anopheline species) but both sexes can also assemble near emergence sites or around vertebrate hosts (mostly culicine species) [50]. Acoustic signals play an important role in mate selection, but the role of pheromones is less clear and only explored for *Aedes aegypti* [51]. Mating behaviour receives increased attention as a result of the proposed releases of sterile insects (SIT) through irradiation or genetic engineering whereby mate finding and competition are crucial for rapid spread in wild populations [52–56]. Furthermore, successful implementation of SIT requires a large production of sterile males for which insights in mating behaviour can be used to optimize rearing conditions and boost mosquito colonies [57, 58]. Attempts to study the behaviour of nocturnal males using a camera and infrared (IR) light date back to 1974, when it was shown that male responsiveness is closely related to their circadian rhythm [59]. Butail & Manoukis et al. [60–62] were the first to record, analyse and discern interactions between wild mating swarms of male *Anopheles gambiae*. On a different scale, mating behaviour has been studied in confined areas using tethered mosquitoes [32, 63, 64] and follow-up studies in larger arenas with untethered *Culex* [65] and *Anopheles* mosquitoes [66]. These behavioural studies provided further evidence how acoustic signals play a role during mate finding and courtship rather than being an epiphenomenon [67]. Exploiting the flight tones to which males respond opens a new array of techniques to either enhance or disrupt mating success or to capture the mosquitoes for monitoring purposes or as a control tool [57, 68–70].

### Host-seeking

Behavioural research on mosquitoes is dominated by studies on host-seeking. This is not surprising, given the direct link with mosquito nuisance and the mosquito capabilities of vectoring human and animal pathogens. Finding a suitable blood host is critical for the uptake of protein needed for vitellogenesis. During host-seeking, mosquitoes make use of multiple host-derived cues [36, 71–73] and flight strategies vary among different species [74]. Host preference and feeding habits are the main drivers for these different strategies [33].

Studies on the host-seeking behaviour of mosquitoes focus on the role of specific cues and, ideally, how the integration with other cues drives their orientation. Because of the multi-modal integration, the exact role of a single cue is difficult to determine [72]. Over the years, extensive behavioural studies on the role of CO_2_, heat, visual cues and specific host odours relevant for host preference have been performed. Outcomes of such studies were often quantified based on responses to a trap and/or by personal observations [75–79]. However, insects display a sequence of behaviours before ending up inside a trap and responses to specific cues that initiate attraction or landing may be missed [77]. With the development of digital tracking techniques, more detailed studies can now be performed, allowing links to be made between detailed in-flight behaviours and a combination of host cues [71, 72, 80–82]. The spatial scale at which such studies are performed is strongly correlated with the host cues of interest. The role of heat or the effects of contact repellents, for example, are studied in confined spaces such as small insect cages or wind tunnels [80, 82–85]. Responses to CO_2_ in host-seeking activation, source localization and host finding are, on the other hand, studied not only in cages and wind tunnels, but also in (semi-) field settings [72, 81, 86].

We distinguish between fundamental behavioural studies that focus on the host cues (mainly volatiles) that mosquitoes can encounter in the wild, and the cues that may affect these behaviours as a result of interventions. Understanding mosquito responses to host cues encountered under natural conditions is an important prerequisite for the correct interpretation of responses to synthetic attractants and/or repellents. Tracking mosquito behaviour to study the effect of (synthetic) attractants, repellents, insecticide-impregnated bed nets or variations in house improvements has shown its relevance in understanding and improving vector-host interventions [21, 22, 87–90].

Behavioural resistance to toxicants is relevant for estimating the effectiveness of interventions with LLINs or IRS. Such behavioural effects cannot be measured with standard WHO susceptibility tests, whereas behavioural data obtained with mosquito-tracking tools can be included in predictive models for the effectiveness of control strategies [91, 92].

### Oviposition

Responses to oviposition cues depend on the physiological state of the mosquito [73, 93, 94]. Female mosquitoes show a temporary absence in behavioural response to host cues when they are fully engorged with blood [95]. After maturation of the eggs, oviposition cues take over and responses to host cues are restored after the eggs have been deposited [95, 96]. At the oviposition site, *Anopheles* and *Culex* species have been observed to hover above the water and make repeated descents, whereas these behaviours are absent in *Ae. aegypti* [97].

Finding and choosing a suitable breeding site is crucial for gravid female mosquitoes. The water body should retain water long enough for full development of the larvae, contain sufficient nutrients and preferably be free of predators [98, 99]. Disrupting this process of oviposition site selection may be highly beneficial for the implementation of (larval) control methods, either by luring the females to a less-favourable breeding site, e.g. one that is treated with a larvicide, or by luring it into oviposition traps [100–103]. Efficient lures could also benefit the development of monitoring tools [104]. Responses to chemosensory cues that are involved in oviposition-site selection are, however, highly species-specific [105]. In addition to the chemical components, visual cues also affect site selection [106, 107]. The success rate of ‘attract-and-kill’ methods can benefit from behavioural observations to unravel the range at which (chemical) cues can mediate the desired behaviour [108, 109]. Interestingly, oviposition behaviour may also be affected indirectly, for example following the exposure to spatial repellents at an earlier stage of the mosquito life-cycle [110].

Behavioural observations on actual egg laying are limited and most studies focus on the ‘end result’ for choice assays by counting egg distributions in response to certain oviposition cues [111–113].

### Larval behaviour

Mosquito larvae are bounded by the breeding site in which they hatched. Their development and survival depend on factors such as water temperature as well as on intra- and inter-species competition over food and space, cannibalism and predation [114–116]. Behavioural responses of larvae can be triggered by direct physical contact or water movement, fluctuations in light intensity (phototaxis), temperature (thermokinetic) or by chemical stimuli (reviewed by Clements [50]). Recently, responses to chemical cues by larvae have been studied to further unravel the olfactory signalling pathway of mosquitoes [34]. 2D tracking of larvae in a Petri dish demonstrated opposite behaviours when exposed to DEET *versus* yeast. As a follow-up, the thermosensory pathways and larval responses of mosquitoes were studied using a similar setup [35].

Behavioural analyses of mosquito larvae are few, but the previously mentioned studies have improved our understanding of sensory response mechanisms in adult mosquitoes [34, 35] and can contribute to model systems for community ecology studies [115].

### Parasite and pathogen mediated mosquito behaviour

Parasite and/or pathogen infections can affect the energy budget and (flight-) behaviour of mosquitoes and this has consequences for their vector competence [117–119]. In fact, parasites and pathogens can manipulate mosquito behaviour directly because of the effects of the infection on the mosquito and indirectly through the cues emitted from the infected hosts [120–124]. Working with infected mosquitoes comes with an extra experimental challenge, as the study organisms should not pose a risk to the local environment and their inhabitants. A number of controlled studies demonstrated the role of mosquito infections and how behaviours may be affected to enhance further transmission of the pathogen [125–130]. Studies with *Plasmodium* infections have demonstrated altered feeding behaviours of mosquitoes [131]. Although there is growing evidence that parasite/pathogen-infected mosquitoes express a modified behavioural response, data on actual flight performance of infected mosquitoes are scarce [132–134].

Infections with entomopathogenic fungi used as a biopesticide, whether or not in combination with *Plasmodium* infections, have also demonstrated to have an impact on flight behaviour and host location [135–139].

On another level, *Wolbachia* infections, which have the potential to disrupt pathogen transmission cycles, have been shown to affect mating success, probing behaviour and increased locomotor activities in *Ae. aegypti* mosquitoes [140–144].

## Insect flight tracking: the state of the art

### Image tracking

Mosquito flight recordings were initially processed manually, by describing or quantifying the recorded behaviours [59, 78, 145]. More accurate quantification was conducted by manually digitizing the recorded images: a laborious task, pointing the subject of interest (the mosquito) frame-by-frame. For 3D analysis, the process had to be done twice with a minimum of 2 × 25 frames per second (fps), requiring 50 mouse-clicks for one second of video recording [24]. Machine-vision technology has developed rapidly and with the increase of computer power, development of high-resolution cameras, the possibilities for automated tracking of mosquito behaviour became available [146]. In short, the technology starts with acquiring images from cameras which are processed using specific thresholds to identify the object (insect) of interest. The thresholds mainly rely on differences in contrast of the image. Calibration of the set-up is critical, especially if data points from two different cameras will be merged for 3D reconstruction of the flight paths. Generally, the direct linear transformation (DLT) method is used [147], in which sets of 2D coordinates obtained from the cameras are linked to the known 3D coordinates of markers on a calibration frame. Depending on the lens type used, lens distortion correction is needed. To date, software with codes for automated tracking can be found online as freeware [148–150]. Other software packages that cover automated tracking, and sometimes 3D reconstruction, are mostly custom-made solutions and some have evolved to an add-on tool next to commercially available motion analysis systems (idTracker, Trackit 3D, Ethovision Track3D) [27, 151, 152]. Overall, the technical development has led to a variety of tracking systems to choose from, a reduction in costs and an increase in the portability of recording set-ups. Advantages and disadvantages of quantification techniques have recently been summarized in a non-exhaustive list by Poh et al. [153]. It is remarkable that the table lists a variety of techniques that are described as either ‘complex to set up’ or ‘cover a small area of observation’ indicating that there is still much room for improvement. Aspects that need to be taken into account to improve tracking systems are discussed below.

### Lighting and contrast

Successful tracking relies on a clear contrast between the object of interest and the background. The insect is either illuminated against a dark background, or it is depicted against a brighter background. Light conditions of the experimental set-up should not interfere with the natural day-night rhythm of the studied mosquito. For *An. gambiae,* it has been suggested that filming with infrared light at λ > 900 nm does not alter the visual environment of the mosquito [154, 155]. Filming at night requires near-infrared lighting so that the reflection of the light on the mosquito wing is caught by IR-sensitive cameras and is visible against a dark background. A drawback here is that most background materials/environments also reflect the IR-light, causing unwanted bright areas that make tracking difficult or impossible. The use of black polycarbonate as a background solves this problem [80]. Black fibreglass netting and ‘blackboard paint’ can serve as alternatives, especially when the contrast of uneven surface areas has to be improved. Another approach is to backlight the cameras so that the mosquito appears as a silhouette in the recorded image [22, 155]. Careful evaluation of the experimental requirements is needed to select optimal lighting solutions. This can be extra-challenging under (semi-) field conditions.

### Multiple object tracking

From the start of the development of automated tracking techniques, scientists searched for solutions to track multiple objects. Occultations are a challenge and in the case of mosquito flight studies, confined arenas are needed to prevent individuals from flying out of sight, with the risk of being confused with other individuals upon returning to the arena. The best tracking results are currently established with tailor-made (MATLAB) tracking codes [60, 72, 151]. Angarita-Jaimes et al. [156] extended the volume of their imaging system by connecting two cameras. This system is able to track multiple mosquitoes within 2.0 × 1.2 × 2.0 m, where it should be noted that the presented parameters on mosquito velocity are based on 2D data. Another way to enlarge the area of interest is by using pan-tilt cameras [27, 157]. By adding extra cameras, provided that they are properly synchronized, occultations can be reduced and additional accuracy and details of flight tracks can be obtained.

### Track the beat

Another method of ‘keeping track’ of mosquitoes is by exploiting their wing beats. When the sound that mosquito wings produce is used to record the activity or passage of mosquitoes, we refer to this as acoustic tracking. Literature on responses of mosquitoes to sound is scarce, but dates back to 1949 [158]. Acoustic tracking has the potential to be used as a relatively cheap method to monitor behavioural activity of mosquitoes. Sound sensors are generally cheaper than image sensors and there is no additional lighting equipment required. Also, the amount of incoming data is smaller compared to image tracking. Differences in wingbeat frequencies can be used to classify individual mosquitoes to species complex level; however, overlap in frequencies does occur [159–161]. Field tests report a major challenge in filtering ambient noise [162], although a recent study using mobile phones as acoustic sensors managed to filter this noise and mosquito identification was succesful [163]. More promising seems the development of opto-acoustic tracking, making use of break-beam technology. When mosquitoes pass a set of infrared emitters and receivers, the interrupted beam is used as an electronic signal by the receiver [164, 165]. Species identification using this technique is challenging, but possible by analysing wingbeat patterns and proof of principle was already reported in 1986 [166, 167]. Automated identification is now reported based on wing movement or wing shape- and pattern analysis and accuracy levels are on the rise [168–172].

### Mosquito behaviour at a glance

Using tracking technology as discussed above, several studies have described the behaviour of mosquitoes during different life stages. We are not aware of studies on oviposition behaviour or plant feeding that have incorporated tracking techniques, but such studies could give additional information on the approach strategies of mosquitoes and measure responses to the provided cues in a (semi-) natural environment [31, 99]. Butail et al. [60] created a 3D reconstruction of a mating event of wild *An. gambiae* mosquitoes, a critical step to characterise the trajectories of both male and female mosquitoes before successful mating occurs. Tracking the behaviour of larvae revealed that thermosensory responses are comparable to those of adult mosquitoes, which may have indirect implications for adult mosquito vectorial capacity, triggered at the larval stage [35]. The same technique was used to demonstrate that larvae show an altered behaviour after exposure to the mosquito repellent DEET [34]. In studies on host-seeking female mosquitoes, Spitzen et al. [80] and McMeniman et al. [72] described how responses to specific host cues are integrated and evoke completely different flight patterns, compared to treatments where cues such as heat or CO_2_ are lacking, or cannot be sensed by mutant mosquitoes. Several studies incorporated mosquito-behavioural analysis in more applied settings directed at interventions with mosquitoes as target. These studies focus on the approach of mosquitoes to host cues in different settings. The obtained information is used to evaluate the effects of the intervention technique [22, 24], or to provide ideas on how and where to implement interventions [90, 136]. The parameters selected to interpret the behaviours of interest show many similarities across studies. Spatial-temporal distributions reveal the relative attractiveness, and thus importance, of the (host-) cues studied. The change in velocity of the insect is closely related to the distance from and intensity of the stimulus, and is a measure of orthokinesis. In addition, the velocity parameter is used as an indicator of the insect nearing mechanical barriers, e.g. when mosquitoes approach a house, bed net or host. Change of headings, the intensity of convoluted pathways, sometimes expressed as the number of turns, are closely related parameters and linked to whether mosquitoes are, or have been, tracking odour cues. At close range, locomotor activity or mobility thresholds can be used to determine whether a mosquito is still in flying modus or has landed and is sitting on or near the target. This parameter is also valuable in 2D tracking systems where accurate estimates of flight velocities are lacking. Setting the mobility threshold should be carefully evaluated, as automated tracking can also produce ‘movements’ while the insect is actually sitting still and the deviation is caused by slight shifts in illuminated pixels [21]. The latter can cause interpretation errors, especially when filming under low resolution.

### Data evaluation and considerations before taking off

The tracking data that are generated need careful evaluation before any meaningful interpretations of the observed behaviours can be made. Occasional tracking errors, for example caused by reflections of IR on interfering objects, should be filtered out to avoid inaccurate data on flight parameters. Such filters can be based on previously-reported maximum flight velocities. Missing data, for example caused by lack of resolution or lighting conditions, can be fixed using interpolation. This avoids unnecessary cuts in tracks that would otherwise be appointed to multiple individuals or flight events. Data interpolation seems especially acceptable for set-ups where more than one camera is used, when missing data is only replaced in cases where the other camera ‘confirms’ the actual continuation of the track [24, 80].

Analysing the average value of measured flight parameters over an entire track may not explain the observed behaviour correctly. For example, the flight speed of host-seeking mosquitoes can drastically change when they lose track of an odour or when they come close to their target [24, 80, 84]. This becomes apparent when the data are divided in sections or bins with certain increments from the target stimuli. With conventional recording speeds (25 fps), however, the number of data points within these smaller sections is limited; this has consequences for the power of the statistical analysis. Recording with higher frame rates solves this problem. For multiple object tracking, high frame rates help to minimize the likelihood of incorrect assignments of individual mosquitoes, as the typical mosquito displacement between frames becomes smaller than the spacing between two individuals [156]. High-speed recording, however, comes with other limitations since it requires extra illumination, computer processing speed and data storage.

Filming with high-resolution cameras at high frame rates generates large data files. When recording gigabytes per minute, data storage becomes the limiting factor for the duration of the behavioural experiment. Real-time tracking would be the solution here, where ‘only’ the *x*,*y*, (*z*) positions of the insect are stored for further analysis and not the video itself. However, the tracking code used should be verifiable using sample videos [173, 174].

## Future perspectives

### Integration of spatial observations with fundamental studies on flight dynamics

Recent studies on the flight kinematics and aerodynamics of mosquitoes, together with the spatial data reviewed above, throw new light on the way mosquitoes move through their environment [29, 175, 176]. Compared to other similarly-sized insects, mosquitoes fly with exceptionally high wing beat frequencies and low amplitudes [175], and after taking a blood meal they can escape from their host without being noticed [176]. Although studying the aerodynamics of mosquito flight often requires an experimental set-up that is highly confined in space, it reveals the physical possibilities and limitations of mosquito flight. Integrating the technical expertise and obtained knowledge on biomechanics with the expanding information on flight behaviours observed in the (semi-) field can accelerate the innovation of vector control tools, for example by fine-tuning fan-powered traps or manipulation of mating behaviour.

### How to exploit mosquito behaviour for surveillance and intervention

With the rapid development and increased availability of tracking hardware and (open-source) software, scientists should consider what system(s) are required to answer their research question(s). This seems obvious, but in the field of mosquito research we distinguish between a focus on fundamental questions on flight behaviour and a more applied approach, where flight data are used for innovation purposes, surveillance, or measuring the effectiveness of intervention strategies. For the applied questions, 2D data are often sufficient and this can drastically reduce the budget required, decrease the amount of incoming raw data and thereby minimize the time required for answering the initial question. For example studies analysing number of landings, or amount of time spent on (insecticide-) treated surfaces, or whether mosquitoes pass certain intervention barriers can benefit from 2D tracking solutions [21, 87, 177]. However, when detailed data on the approach of mosquitoes to certain targets, or responses to particular cues is requested, 3D data are a prerequisite because of their typical convoluted flight patterns, both horizontally and vertically [72, 80].

Our understanding of behavioural repertoires of mosquito vectors during their life-cycle, whether infected or not, can be exploited for the development and effective implementation of novel monitoring and control tools (e.g. [16, 133, 178]). Behavioural data can be added to data obtained from conventional monitoring tools such as traps, resting catches and human landing collections, in order to validate models on malaria transmission [179, 180]. Tracking techniques can be combined with trapping techniques or even replace traps as a surveillance tool to assess mosquito abundance or activity. For the monitoring of species abundance, or measuring the activity of mosquitoes around human dwellings, there is no need to analyse the full behavioural repertoire. The break-beam technology that exploits specific wing beat characteristics can be advantageous here, especially when combined with a timer to register circadian rhythms [164, 165, 171, 181]. Whereas the use of microphones seems inapplicable for larger arenas because of background noise, the use of opto-acoustics to measure activity and classify mosquitoes that enter houses (e.g. *via* eaves or eave tubes) or traps may provide an easier-to-install and cheaper method than filming. Even if species recognition is imperfect, the technique can still provide data on flight activity on a temporal scale. Given their size, it is unlikely that (harmonic) radar systems will become available to follow long-range movements of mosquitoes [182–184]. Aerial sampling with balloons at 40–250 m above ground level revealed that a significant number of mosquitoes exploit wind streams to migrate at high altitudes (Tovi Lehmann, personal communication). Such studies can help to explain seasonal (re-) appearance of mosquito species and predict the need for continuous vector control measures. Mark-recapture studies form another alternative to measure (migration) movements, with the challenge being to capture the wild individual in the first place so that it can be marked [185]. The latter can be solved by using the stable isotope method, with which larvae can be marked and re-captured as adults [186]. Mark-recapture studies will not reveal details on movements between point A and B, but can give valuable information on mosquito dispersal.

## Conclusions

The implementation of vector intervention tools, as part of the Global Vector Control Response strategy launched by the WHO [20], comes along with questions on the effectiveness and possible behavioural adaptations of mosquitoes to such tools. For example, the shift towards outdoor transmission by behaviourally resistant mosquitoes requires adjustments in intervention techniques [180]. Recent developments in insect tracking technology are promising for further implementation in the field and are expected to provide the necessary feedback and estimations of mosquitoes that pass through holes, eaves, windows and doors [89, 90, 187, 188]. Interventions may have an effect on airflow coming from houses and quantifying this requires particle tracking of airflows, as reviewed by Fu et al. [189]. More directly, manipulating the airflow using fog dispensers has shown to affect the flight capabilities of mosquitoes and could function as an intervention tool in itself [190]. To date, automated tracking techniques have contributed to our understanding of how the multimodal integration of (host-) cues plays a role in source finding of mosquitoes [72, 80]. The tools have provided information on attraction or repellent modes by studying mosquito behaviour around treated surfaces such as bed nets [21, 22, 191, 192] or around odour-baited traps [24]. Recently, the obtained knowledge led to promising implementations in the field where adding a heat source to an odour-baited trap resulted in a 6.5 fold increase of trap catches of *An. gambiae* compared to traps without heat [193].

## Abbreviations

2D: two-dimensional
3D: three-dimensional
DEET: n,n-diethyl-meta-toluamide
DLT: direct linear transformation
fps: frames per second
IR: infrared
IRS: indoor residual spraying
LLINs: long-lasting insecticide-treated nets
SIT: sterile insect technique
WHO: World Health Organization
